# Shifting From Systemic to Precision‐Targeted Complement Therapies: Opportunities and Hurdles

**DOI:** 10.1002/eji.70231

**Published:** 2026-06-19

**Authors:** Marco Mannes, Wioleta M. Zelek, Leendert A. Trouw

**Affiliations:** ^1^ Institute of Clinical and Experimental Trauma‐Immunology Ulm University Medical Center Ulm Germany; ^2^ UK Dementia Research Institute at Cardiff University, School of Medicine Cardiff University Cardiff UK; ^3^ Department of Immunology Leiden University Medical Center Leiden the Netherlands

**Keywords:** complement system, complement therapeutics, targeted delivery

## Abstract

The landscape of complement therapeutics has significantly broadened in recent years; systemically acting stoichiometric inhibitors against complement proteins across almost the entire complement cascade are now available. However, despite their unquestionable clinical success, several limitations remain, including increased infection risks, loss of physiological functions, and clinically observed breakthrough events. In addition, many complement diseases require lifelong treatment, further amplifying the overall healthcare burden and motivating the development of alternative concepts for more precision‐based therapies. Given that many complement‐associated diseases primarily manifest in specific body compartments, directing complement intervention to an organ, tissue, or even a particular cell type represents an important goal. In this article, we briefly delineate the status of approved complement therapeutics and review preclinical progress in emerging concepts. We highlight several key properties that are essential for achieving targeted complement therapies: administration routes, tissue/cell penetration, specificity, and the mode of action. In addition to approaches aimed at dampening complement activation, we outline strategies designed to specifically activate complement locally, for example, in the context of cancer. Together, these insights underscore the growing potential of next‐generation complement therapeutics to achieve more precise and effective clinical outcomes.

## Graphical abstract

Complement therapeutics have expanded considerably, but systemic inhibitors remain limited by infection risks, breakthrough events, and loss of physiological functions. Emerging targeted approaches aim for organ‐, tissue‐, or cell‐specific modulation of complement activity, potentially offering greater precision while reducing treatment burden and preserving beneficial complement functions.

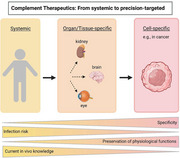

## 1 Introduction

The complement system is a network of more than 40 plasma and cell‐bound proteins that continuously patrol the bloodstream in a quiescent state, sensing the intravascular environment for molecular patterns that differ from healthy host surfaces. These include not only pathogens (i.e., pathogen‐associated molecular patterns, PAMPs), but also altered host surfaces (e.g., apoptotic cells) and immune complexes, all of which must be cleared to maintain homeostasis [1]. Traditionally, the blood‐borne complement system is described as comprising three canonical activation pathways. Two of these, the classical and lectin pathway (CP and LP), are triggered by specific recognition events, whereas the alternative pathway (AP) is characterized by a continuous, low‐grade, self‐activating mode. Activation of these pathways results in opsonization, triggers inflammation, and can mediate target cell lysis. However, accumulating evidence indicates that the complement system is far more complex than just the three activation pathways and is also involved in extensive pathway crosstalk [2], local complement production [3, 4], and additional activation mechanisms beyond these three canonical pathways [5, 6]. Moreover, evidence for cell‐intrinsic and intracellular complement has added a new dimension to complement research, suggesting that complement proteins may also perform functions distinct from their traditional extracellular roles (reviewed in [7, 8, 9]).

Although the complement system plays essential roles in protection from infections and numerous physiological processes, it also contributes to the pathology of a wide array of human diseases. In these diseases, pathological complement activation is triggered by either excessive activation or by insufficient regulation. Excessive complement activation is observed and is thought to contribute to disease outcomes across a wide range of conditions, including both acute and chronic disorders, as well as common and ultra‐rare diseases. Therefore, complement has emerged as an attractive therapeutic target, as its enzymatic cascade can be readily modulated and is, therefore, highly druggable.

Complement‐targeting drugs are currently primarily utilized for rare diseases (e.g., paroxysmal nocturnal hemoglobinuria [PNH] and atypical hemolytic uremic syndrome [aHUS]), although the range of therapeutic options continues to expand. The use of complement inhibitors in more common diseases is limited and remains constrained by high costs, concerns regarding benefit‐risk balance, increased susceptibility to infections, and the potential loss of physiological complement functions. Nevertheless, the FDA approval of proximal (pegcetacocplan; [10]) and terminal (avacincaptad pegol; [11]) complement inhibitors for the treatment of geographic atrophy (GA) secondary to age‐related macular degeneration (AMD), the third leading cause of vision impairment [12], represents an important step in this direction. However, local application of complement‐inhibitory drugs is only feasible for specific locations such as the eye and inflamed gums [1, 13]. Addressing challenges such as costs, safety, and alternative routes of administration, overcoming biological barriers (e.g., blood–brain barrier [BBB], blood–retina barrier [BRB]), and delivering drugs specifically to sites of complement dysregulation, will be critical for expanding complement inhibitors to more common diseases.

In this review, we summarize currently approved inhibition strategies and discuss the factors limiting their broader clinical application. Building on insights from clinical use, we outline perspectives for next‐generation complement therapeutics. In addition to complement inhibition, we also discuss strategies aimed at activating complement, which may offer therapeutic potential in diseases that could benefit from localized complement cascade activation, such as cancer.

## 2 Complement Inhibition: From the First Step to Where We Are Now

As early as the end of the 1960s, Cochrane et al. [14] demonstrated that in vivo complement depletion markedly reduced tissue injury in various immunologic reaction models. They used cobra venom factor (CVF), which is a potent complement‐activating protein that ultimately induces systemic complement consumption and renders the cascade unresponsive to subsequent stimuli [15]. This experiment provided one of the first direct demonstrations that modulation of complement activation can ameliorate disease pathology. Thus, CVF functioned not only as an experimental reagent, but also as a conceptual proof‐of‐principle that complement is a therapeutically relevant target, providing a strong rationale for complement‐modulating therapies.

**FIGURE 1:**
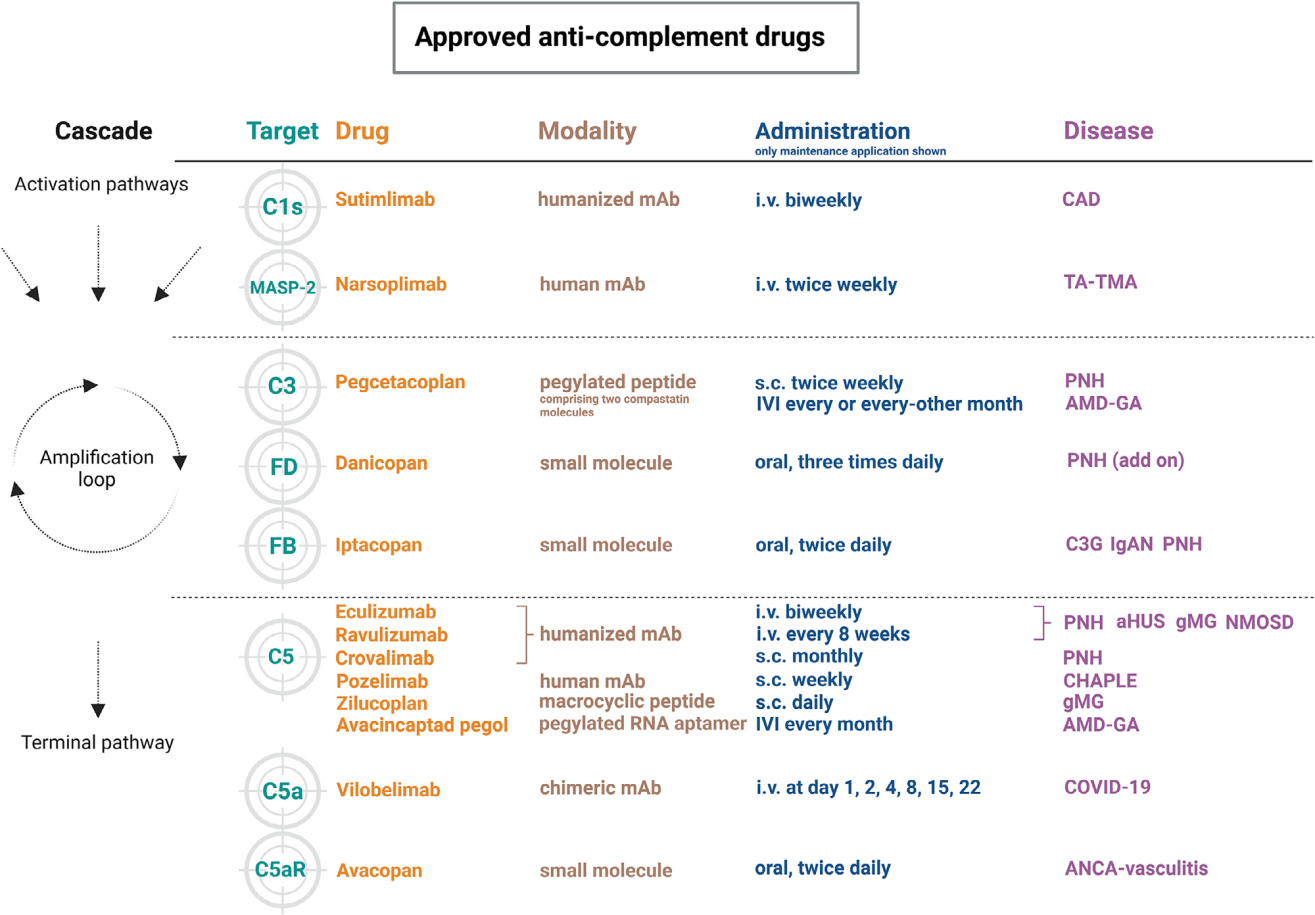
Approved drugs targeting the complement system. List of currently approved drugs targeting the complement system, including molecular targets, drug names, modalities, route of administration, and approved disease indications. mAb: monoclonal antibody, i.v.: intravenous; s.c.: subcutaneous; IVI: intravitreal injection; CAD: cold agglutinin disease; TA‐TMA: transplant‐associated thrombotic microangiopathy; PNH: paroxysmal nocturnal hemoglobinuria; AMD‐GA: geographic atrophy secondary to age‐related macular degeneration; C3G: C3 glomerulopathy; IgAN: IgA nephropathy; aHUS: atypical hemolytic uremic syndrome; gMG: generalized myasthenia gravis; NMOSD: neuromyelitis optica spectrum disorder; CHAPLE: complement hyperactivation, angiopathic thrombosis, and protein‐losing enteropathy; ANCA‐vasculitis: antineutrophil cytoplasmic autoantibody‐vasculitis.

Half a century later, we now count more than a dozen approved complement inhibitors across eleven complement‐driven diseases (Figure 1). In this section, we restrict to a brief description of the molecular targets of currently approved complement inhibitors and refer to excellent recently published reviews for more details regarding the underlying diseases [16, 17].

One of the earliest attractive targets for therapeutic intervention was C5 [18], as it represents a convergence point of all proximal activation pathways and gives rise both to the potent anaphylatoxin C5a and to the membrane attack complex (MAC). The pioneering drug eculizumab, a monoclonal antibody targeting C5, emerged at the end of the last century following screening of murine antibodies for their capacity to block MAC formation. Afterwards, humanization was performed for clinical use [19, 20, 21].

To date, eculizumab has been approved for the treatment of several disorders driven by dysregulated terminal pathway (TP) activation, including PNH [22], aHUS [23], generalized myasthenia gravis (gMG) [24], and neuromyelitis optica spectrum disorder (NMOSD) [25]. Despite its clinical success, long‐term experience with eculizumab has revealed limitations, including a relatively short plasma half‐life necessitating biweekly intravenous administration. In addition, reports described a reduced efficacy in a subset of patients carrying specific C5 polymorphisms, most notably the R885H variant, which affects approximately 4% of the Japanese population [26]. These considerations have driven the development of next‐generation C5 targeting therapeutics. Ravulizumab, a modified variant of eculizumab [27], has largely replaced it in several indications due to improved pharmacokinetic properties. Four engineered amino acid substitutions enable pH‐dependent dissociation of C5 in the endosome and enhanced FcRn‐mediated recycling of the mAb, resulting in prolonged half‐life (∼50 days compared with ∼11 days for eculizumab) and extended dosing intervals of up to 8 weeks without compromising clinical efficacy [27, 28, 29]. In fact, ravulizumab is now approved for the same indications as eculizumab [29, 30, 31, 32, 33].

More recently, the approval of crovalimab for PNH has further expanded therapeutic options. This includes treatment of patients who are resistant or inadequately responsive to eculizumab, as crovalimab binds to a distinct epitope on C5 and retains activity in the R885H variant [34]. In addition, its subcutaneous route of administration with a 4‐week dosing interval provides an alternative to the previously established intravenous therapies, enabled by the engineered pH‐dependent “sweeping” monoclonal antibody (mAb) technology, as employed in ravulizumab [35]. Direct comparisons between crovalimab and eculizumab have demonstrated comparable efficacy and safety in the treatment of PNH [36].

Another antibody that blocks intact C5 is the fully human mAb pozelimab, which was approved for the treatment of complement hyperactivation, angiopathic thrombosis, and protein‐losing enteropathy (CHAPLE) disease, a rare inherited disorder caused by deficiency of the complement regulatory protein CD55 [37].

In addition to these antibody‐based therapies, two non‐antibody modalities targeting C5 have been approved for clinical use. Zilucoplan, a macrocyclic peptide inhibitor, is approved for the treatment of gMG [38]. Avacicaptad pegol, a pegylated RNA aptamer, is approved for the treatment of geographic atrophy secondary to AMD [11].

Identification of the small cleavage product of C5, C5a, as a key pathological driver has shifted therapeutic focus toward the C5a/C5aR1 axis in specific diseases and conditions. In antineutrophil cytoplasmic antibody (ANCA)‐associated vasculitis, the peptide‐based C5aR1 antagonist avacopan has been approved for oral clinical use [39]. Vilobelimab, a mAb that targets C5a itself rather than its receptor, has received emergency use authorization for the treatment of COVID‐19 in patients within the first two days following initiation of invasive mechanical ventilation or extracorporeal membrane oxygenation [40].

Although TP inhibition has transformed the management of several complement‐mediated disorders, it does not directly suppress upstream complement dysfunction. Diseases in which C3‐mediated effector functions contribute to pathology—either independently or particularly in the context of terminal blockade—benefit from more upstream intervention. For example, C5 blockade in PNH increases the proportion of C3‐opsonized erythrocytes [41], thereby enhancing their clearance in extravascular compartments and leaving some patients transfusion‐dependent [29]. This limitation has driven the development of proximal inhibitors, culminating in the approval of the compstatin‐based, pegylated C3 peptide inhibitor pegcetacoplan for the treatment of PNH in 2021 [42], followed by its approval for GA secondary to AMD [10]. However, because C3 occupies a central position in the complement cascade, its inhibition results in broader complement suppression, and long‐term safety considerations such as infection risk and impact on general physiology require careful evaluation.

In addition to directly targeting C3, therapeutic strategies have been developed to inhibit key components of the alternative pathway amplification loop. Danicopan, an oral small‐molecule factor D (FD) inhibitor [43], is approved as add‐on therapy to standard‐of‐care C5 inhibitors (eculizumab and ravulizumab) in PNH, particularly for patients with clinically substantial extravascular hemolysis [44]. Clinical data demonstrate that the combined proximal and terminal inhibition strategy significantly improved hematological parameters without additional safety concerns [45]. However, danicopan is currently approved only as an add‐on therapy, reflecting that FD inhibition alone may not provide sufficient complement control, in part due to rapid consumption of the target within the amplification loop.

More recently, the molecular target of FD, Factor B (FB), has emerged as another therapeutic option. The orally administered FB inhibitor iptacopan, given twice daily, has demonstrated efficacy in PNH and in the renal diseases IgA nephropathy and C3 glomerulopathy [46, 47, 48, 49]. In contrast to danicopan, iptacopan is used as monotherapy across those indications.

For diseases in which a specific activation pathway predominantly drives pathology, inhibitors targeting the initiating steps of the complement cascade have been developed. The humanized monoclonal antibody sutimlimab neutralizes C1s in cold agglutinin disease (CAD), thereby selectively inhibiting the classical pathway and preventing IgM autoantibody‐mediated erythrocyte destruction [50]. Analogous to C1s inhibition in the classical pathway, the fully human monoclonal antibody narsoplimab targets mannan‐binding lectin serine protease 2 (MASP‐2), thereby blocking activation of the lectin pathway. Recently, narsoplimab received FDA approval for the treatment of hematopoietic stem cell transplant‐associated thrombotic microangiopathy (TA‐TMA) after demonstrating improvements in organ function and survival outcomes [51, 52, 53]. However, the therapeutic effect of pathway‐specific inhibitors is expected to depend on the extent to which the respective pathway contributes to overall disease pathology.

Taken together, the approval of complement‐targeted therapies has revolutionized the management of rare complement‐driven diseases and substantially improved patients’ quality of life. Nearly two decades after the first approval, numerous additional agents have entered the market, greatly expanding therapeutic options for clinicians, particularly in PNH. The current therapeutic repertoire enables intervention at virtually every level of the complement cascade, including the initiating pathways, the amplification loop, and the TP.

Despite this scientific, industrial, and clinical success, the broader application to more common diseases, in which complement activation is a major contributor but not the sole pathogenic driver, remains limited. To expand the therapeutic use of complement inhibitors, several key questions need to be addressed: What are the current limitations that hinder the application of complement inhibitory therapeutics in such settings? What lessons can be drawn from observational studies, experimental studies, and clinical experience to guide the development of next‐generation complement therapeutics?

## 3 Factors That Might Limit Broader Use of Currently Approved Inhibitors

One important factor restricting their wider use is the substantial economic burden associated with these (orphan) complement therapeutics. A combination of development, manufacturing, and market factors has positioned these agents among the most expensive drugs in modern medicine. For example, eculizumab held a market monopoly in PNH for more than a decade and is associated with annual treatment costs exceeding US$500,000 per patient [54]. As eculizumab does not correct the underlying hematopoietic stem cell abnormality in PNH, patients typically require lifelong therapy, resulting in a substantial cumulative economic burden. The introduction of alternative C5‐ and proximal inhibitors has, however, created more cost‐effective therapeutic options. Ravulizumab has been reported to improve health‐related quality of life in patients with PNH while demonstrating a favorable cost‐saving profile compared with eculizumab, suggesting savings of ∼25% depending on the healthcare setting [55]. Furthermore, in a complement treatment‐naïve PNH cohort, a cost‐effectiveness model estimated considerable lifetime savings (∼ 30 %) and additional quality‐adjusted life years with pegcetacoplan compared with both eculizumab and ravulizumab [56]. Similarly, a cost‐per‐responder analysis in both C5‐inhibitor‐experienced and treatment‐naïve patients demonstrated higher response rates and lower costs per responder for iptacopan compared with eculizumab and ravulizumab [57]. In addition, two eculizumab biosimilars were approved in 2024 for PNH and aHUS, with projected annual savings of approximately 30 % compared with the originator product [58]. While these developments indicate a positive trend toward improved economic sustainability, overall treatment costs remain high.

On top of this, a central challenge of systemic anticomplement therapies arises from the intrinsic biochemical properties of complement proteins. Several complement components are present in exceptionally high plasma concentrations, most notably C3, which circulates at levels of approximately 1–1.5 mg/mL [59]. Effective systemic inhibition of such abundant targets requires sustained drug concentrations to achieve sufficient target engagement, which often necessitates frequent dosing to maintain adequate pharmacokinetic exposure. In clinical practice, this has prompted discussion of alternative strategies in selected patients, including treatment interruption or switching to longer‐acting complement inhibitors to reduce treatment burden while maintaining disease control (e.g., [60]). In contrast, other complement factors, such as Factor D, are characterized by comparatively low plasma abundance but exhibit rapid turnover and high resynthesis rates, estimated to be more than 1 mg/kg/day in humans [61]. This continuous production, together with rapid replenishment, can restore functional activity despite ongoing pharmacological inhibition, which can ultimately attenuate drug efficacy.

Another concern that might limit the broader application of systemic complement‐targeted therapies is the potential impairment of host defense against infections, as well as a possible increased long‐term risk of autoimmune diseases. These concerns are supported by observations from individuals with genetic deficiencies of complement components throughout the cascade [62]. Deficiencies of the TP are associated with increased susceptibility to *Neisseria* species, whose clearance critically depends on MAC formation [63]. Deficiencies affecting upstream pathways, for example, C3, are associated with a broad susceptibility to infections due to impaired opsonization and release of anaphylatoxins. Accordingly, treatment with eculizumab has been associated with an approximately 1000‐fold increased risk of meningococcal infection, necessitating vaccination before therapy initiation. Although vaccination substantially reduces the risk, it does not eliminate it entirely [64], and prophylactic antibiotic treatment is therefore often recommended for patients receiving C5 inhibitors [65]. For pegcetacoplan, vaccination requirements are broader and include immunization against *Neisseria meningitis*, *Streptococcus pneumoniae*, and *Haemophilus influenzae type b*, aiming to provide optimal protection against encapsulated bacteria.

Insights from studies of autoimmune diseases further demonstrate that genetic deficiencies, particularly of the CP, constitute strong risk factors for the development of systemic lupus erythematosus (SLE) [66]. Although no increased incidence of autoimmune disease was observed following sutimlimab administration in the CARDINAL trial evaluating its efficacy in CAD [50, 67], the theoretical risk of inducing autoimmunity in a long‐term perspective based on its mode of action is acknowledged in the FDA prescribing information [68]. Careful evaluation of the benefit‐risk ratio is therefore warranted when considering complement inhibition strategies in more common diseases.

A related concern arises from the potential loss of essential physiological functions of the complement system. Complement factors contribute to regenerative and homeostatic processes, including the clearance of apoptotic cells and immune complexes. In murine models, direct involvement of complement activation products in liver regeneration and fracture healing has been demonstrated [69, 70, 71]. To what extent these findings are translatable to humans remains unclear; however, this possibility should be taken into consideration.

## 4 Lessons From Approved Complement Inhibitors

Beyond these general considerations, clinical experience from real‐world patient data has revealed unexpected observations. All currently approved complement inhibitors act in a stoichiometric manner, meaning that they bind their target in a fixed ratio with the aim of completely neutralizing the target and, consequently, its downstream effector functions.

However, preclinical and clinical observations suggest that this theoretical model does not always fully translate into practice. The most compelling evidence comes from patients treated with C5 inhibitors. Detailed longitudinal analyses of eculizumab‐treated PNH patients have documented breakthrough hemolytic events that cannot always be explained by pharmacokinetic factors alone [72]. TP activation has been observed despite apparently adequate pharmacological C5 inhibition, particularly during episodes of complement‐amplifying conditions such as infections or inflammation. In these so‐called pharmacodynamic breakthrough episodes, the inhibitory capacity of the drug can be overcome. Mechanistically, this phenomenon has been attributed to increased complement opsonization, which promotes C5 activation through conformational changes in a nonproteolytic manner [73]. These findings illustrate that the complement system can employ escape mechanisms that allow activation to proceed despite stoichiometric blockade. Regarding proximal inhibition, breakthrough events could theoretically become even more severe, as overcoming the blockade would progress to activation of the unprotected terminal pathway [74]. Initial clinical trial data suggested less frequent breakthrough hemolysis events under pegcetacoplan therapy compared with eculizumab [42]. Recently, efficacy assessment of pegcetacoplan in PNH over a 3‐year follow‐up period revealed that nearly 30% of patients experienced a laboratory‐confirmed breakthrough [75]. However, so far, long‐term clinical experience with proximal inhibitors remains limited, and the available data are currently insufficient to determine the relative contributions of pharmacokinetic and pharmacodynamic breakthrough.

Taken together, as emphasized earlier [76], complement inhibition has revealed unexpected complexities that were not previously anticipated. In particular, it has uncovered parallel, noncanonical activation or progression pathways that may limit stoichiometric inhibitor efficacy under specific conditions.

## 5 Compartmentalization of Complement Diseases

Apart from locally administered therapies used for the treatment of AMD‐GA, all currently approved complement‐targeting drugs act systemically. However, it is increasingly recognized that most complement‐driven diseases primarily manifest within specific organs or tissues, whereas the resulting activation products disseminate via the bloodstream and often form the basis for clinical assessment.

For example, aHUS primarily affects distinct endothelial sites within the kidney, while gMG targets a highly specific anatomical region at the neuromuscular junction. Even diseases traditionally considered systemic, such as PNH, are functionally localized to defined anatomical structures, specifically the surface of erythrocytes.

Furthermore, the classical dogma that complement components are derived exclusively from hepatic synthesis has been revised. Multiple disease settings, including acute and chronic inflammatory conditions, can substantially alter local expression levels of complement components that are critical for disease progression [77, 78, 79]. Endothelial, epithelial, and immune cells have been shown to produce sets of complement proteins [3]. Transplantation studies using kidney isografts from C3‐deficient mice implanted into C3‐sufficient recipients demonstrated reduced tubular injury and renal failure, indicating that locally produced C3, rather than circulating C3, acts as the principal disease driver [80].

Together, these perspectives and findings highlight a central paradox in complement therapeutics: despite detailed knowledge of disease‐specific sites of complement activation and local complement production, currently available therapeutic strategies predominantly rely on systemic inhibition. This mismatch is particularly evident in anatomical compartments that are largely segregated from systemic circulation, such as the eye and the brain. Consequently, strategies enabling local or compartment‐targeted complement modulation are increasingly recognized as an important next step to improve therapeutic precision while minimizing systemic exposure. Here, we aim to illustrate this challenge by focusing on two major complement disease hotspots and summarize recent advances in anticomplement therapeutic strategies within chosen compartments.

## 6 Two Complement Disease Hotspots: Brain and Eye

**FIGURE 2:**
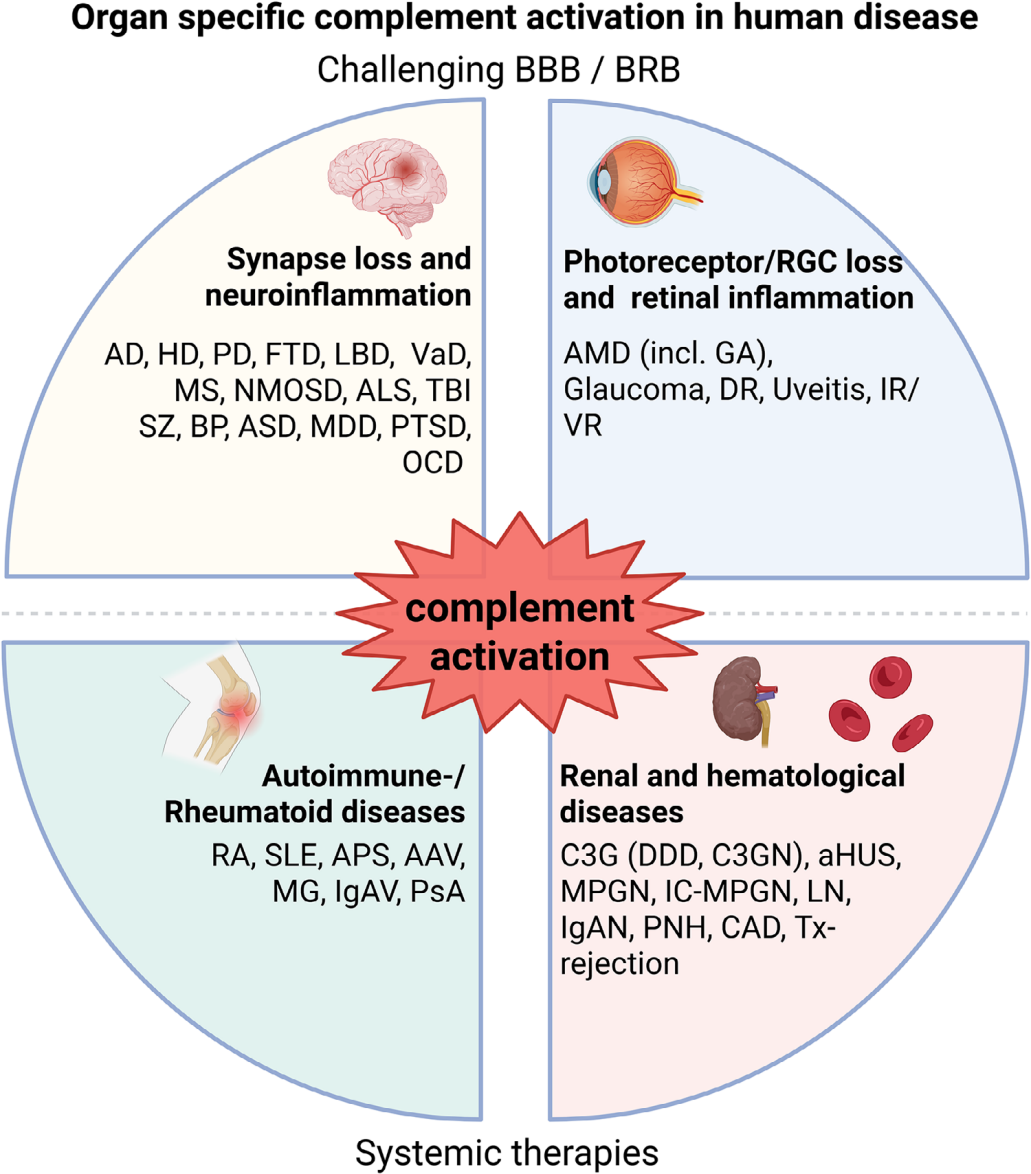
Organ‐specific complement dysregulation and disease. Complement activation is compartmentalized in organs, where local complement activation drives the disease. In the central nervous system (CNS), complement dysregulation contributes to synapse loss and neuroinflammation, implicated in neurodegenerative and neuroinflammatory diseases such as: Alzheimer's disease (AD), Huntington's disease (HD), Parkinson's disease (PD), frontotemporal dementia (FTD), Lewy body dementia (LBD), vascular dementia (VaD), multiple sclerosis (MS), neuromyelitis optica spectrum disorder (NMOSD), amyotrophic lateral sclerosis (ALS), and traumatic brain injury (TBI). Emerging evidence suggests that complement plays a significant role in driving pathology in neuropsychiatric and neurodevelopmental diseases, including schizophrenia (SZ), bipolar disorder (BP), autism spectrum disorder (ASD), major depressive disorder (MDD), posttraumatic stress disorder (PTSD), and obsessive–compulsive disorder (OCD). In the retina, complement activation drives photoreceptor and retinal ganglion cell (RGC) loss and retinal inflammation in age‐related macular degeneration (AMD, including geographic atrophy [GA]), glaucoma, diabetic retinopathy (DR), uveitis, and retinal ischemic/vascular retinopathies (IR/VR). In the joint, immune‐complex–driven complement activation promotes synovial inflammation and tissue injury in diseases such as rheumatoid arthritis (RA), systemic lupus erythematosus (SLE), antiphospholipid syndrome (APS), ANCA‐associated vasculitis (AAV), IgA vasculitis (IgAV), and psoriatic arthritis (PsA). In renal and hematological diseases, complement dysregulation is a central driver of pathology in C3 glomerulopathy (C3G; encompassing dense deposit disease [DDD] and C3 glomerulonephritis [C3GN]), atypical hemolytic uremic syndrome (aHUS), membranoproliferative glomerulonephritis (MPGN, including immune‐complex MPGN), lupus nephritis (LN), IgA nephropathy (IgAN), paroxysmal nocturnal hemoglobinuria (PNH), and cold agglutinin disease (CAD), Tx‐rejection (transplant rejection).

The brain and eye are immune‐privileged, protected by vascular barriers—respectively the blood–brain barrier (BBB) and blood–retinal barrier (BRB), which tightly regulate cellular and molecular trafficking [81]. In both organs, neurodegenerative lesions are local “hotspots” for complement activation; complement proteins (e.g., C1q, C3, C5b‐9) colocalize with Aβ plaques in Alzheimer's disease (AD) and with drusen in AMD [82, 83, 84, 85], driving complement‐mediated tissue injury and chronic inflammation, with downstream consequences including synapse loss and neuronal injury [86, 87, 88, 89]. Complement activation in the brain and retina relies on local production. In the retina, Müller glia and astrocytes are major sources of C3, while C1q is produced mainly by microglia, with Müller glia providing an additional source; reactive glial states further increase complement expression in injury and disease [90, 91, 92, 93, 94]. In the brain, C1q is produced exclusively by microglia, tagging synapses for elimination and initiating CP activation. Microglia are the principal source of most complement proteins in the CNS, although astrocytes contribute to C3 generation, particularly under inflammatory conditions [87, 95]. Complement dysregulation also impacts the neurovascular unit: C1q, C3aR, and C5aR signaling promote leukocyte recruitment, driving inflammation, disrupting tight junction integrity, and contributing to the breakdown of the BBB/BRB [96, 97, 98, 99, 100, 101]. In the retina, compartmentalization extends to the outer BRB (Bruch's membrane/retinal pigment epithelium interface), where complement regulation relies predominantly on Factor H (FH)/Factor H‐like protein 1 (FHL‐1) and Factor I (FI). This outer barrier role may provide a mechanistic explanation for the strong genetic association between the CFH Y402H risk allele and AMD [102, 103, 104, 105]. Complement dysregulation is also evident across a broader spectrum of neurological and retinal disease disorders, as summarized in Figure 2.

Beyond its established immunodefence roles, intracellular roles of complement have been described, where cleavage of C3 and C5 in cell compartments generates bioactive fragments implicated in metabolic and stress‐signaling pathways. Whether such intracellular complement operates within CNS cell compartments to contribute to neurodegenerative pathology remains unknown [106].

Complement therapeutics in retinal disease have advanced rapidly, enabled by local (intravitreal) delivery that circumvents barrier constraints, and by high‐resolution retinal imaging (optical coherence tomography and fundus autofluorescence), enabling precise, longitudinal monitoring of pathophysiology. Currently, two complement therapeutics are FDA‐approved for GA: pegcetacoplan (a PEGylated cyclic peptide C3 inhibitor) and avacincaptad pegol (a PEGylated RNA aptamer that blocks cleavage of C5), although neither has secured equivalent European market approval (pegcetacoplan received a negative CHMP opinion, while the avacincaptad pegol application was withdrawn). Numerous complement agents are also in trials, including C1q blocking mAb (ANX007; NCT03488550, NCT04188015, NCT04656562, NCT06510816; IVT delivered), the FB‐targeting antisense oligonucleotide (IONIS‐FB‐LRx; NCT03815825, NCT03446144; subcutaneous injection), and iptacopan (NCT05230537; oral delivery), which is being evaluated for prevention of progression from earlier AMD stages rather than treatment of established GA. However, not all trials have been successful in GA: notably, trials of FD inhibitors lampalizumab failed to demonstrate clinical benefit in phase III trials, and trials with danicopan were terminated due to insufficient patient benefit [107, 108]. Recent efforts have focused on broader pathway targeting, including C3 inhibition (mAb, IVT; NGM621; NCT04643886) and gene therapy strategies aimed at restoring complement regulation or blocking of the AP and TP, including AAV‐CFI (GT005; NCT03846193, NCT04437368, NCT04566445, NCT05481827) and AAV‐CD59 (JNJ‐81201887/HMR59; NCT03144999, NCT05811351), although several programs have reported limited efficacy or halted development (e.g., NGM621, GT005) [11, 109, 110, 111, 112].

Despite strong genetic and mechanistic evidence linking complement to neurodegeneration, including genetic risk associations such as *CR1* and *CLU* in AD [113, 114] and emerging neuropathological evidence in Huntington's disease (HD) and amyotrophic lateral sclerosis (ALS) [106, 115, 116, 117, 118], the clinical success of complement therapeutics has not yet extended to neurodegenerative brain diseases. Consequently, the clinical translation of these therapies in this field remains at an early stage. Interestingly, systemically administered C1q blocking mAb (ANX005) has entered early‐stage trials in HD and ALS (NCT04514367, NCT04569435). However, early trials blocking C5 cleavage with ravulizumab (NCT04248465) and zilucoplan (HEALEY ALS platform trials, NCT04297683; related registration: NCT04436497) failed to demonstrate clinical efficacy in ALS [119, 120], highlighting the complexity of trial design requiring target selection, delivery, and biomarker development in CNS indications. Notably, ravulizumab is approved for NMOSD (NCT04201262), proving that complement inhibition can succeed in a CNS disease where target engagement is achievable owing to focal BBB disruption.

These examples illustrate that efforts to modulate complement in neurodegenerative disease have to date relied predominantly on systemically administered inhibitors. However, complement activation is largely compartmentalized within the CNS, behind an intact BBB, particularly at early stages of pathology when therapeutic intervention is likely to be most effective. Achieving pharmacologically meaningful CNS target engagement, therefore, represents a central challenge for next‐generation complement therapeutics. Strategies to overcome the BBB include receptor‐mediated transcytosis (RMT) exploiting endogenous endothelial receptors, for example, the transferrin receptor (TfR) [121, 122], to shuttle large biologics such as mAbs, which otherwise achieve only negligible brain exposure (∼0.1% via passive diffusion) [123]. The use of TfR‐shuttle to deliver anti‐C1s (Sanofi) and anti‐C7 blocking antibodies is in preclinical development for Alzheimer's disease (AD) [124]. The brain‐penetrating anti‐C7 antibody achieved CNS target engagement, resulting in reduced neuroinflammation, protection of synapse loss, and improved cognition in the App^NL‐G‐F^ mouse model of AD [124], providing proof‐of‐concept for therapeutic modulation of complement within the brain. Additional approaches include AAV‐mediated gene delivery using CNS‐tropic serotypes (e.g., AAV9, AAVrh10) to enable local therapeutic expression, and nanoparticle and extracellular vesicle cargo carriers [125, 126]. These strategies show promise, achieving meaningful CNS target engagement; however, their translational maturity remains variable and highly context‐dependent, with important considerations in relation to manufacturability, dosing paradigms, and long‐term safety [127, 128]. More invasive interventions, such as intrathecal administration, convection‐enhanced parenchymal infusion, or transient barrier disruption via focused ultrasound or osmotic opening, may be appropriate for acute indications but are unlikely to constitute scalable solutions for chronic neurodegenerative diseases requiring repeated long‐term treatment. The abovementioned strategies and their respective advantages and limitations have been extensively reviewed elsewhere [106, 121, 122]. Successful therapeutic modulation of complement within the brain will therefore depend not solely on target selection, but on integrated pharmacological design encompassing efficient barrier traversal, avoidance of peripheral target engagement (“sink effect”), and sustained parenchymal exposure. In this context, brain‐penetrant small molecules may offer advantages for achieving sustained complement inhibition in CNS diseases.

## 7 Targeting the Right Place: Tissue Access and Molecular Specificity of Complement Inhibition

Tissue penetration represents one of the most important considerations for therapeutics. In this context, it is essential to distinguish between anatomical sites that are readily accessible to plasma‐derived complement components and those in which complement activation is primarily driven by local complement production. So far, the focus of industry has been largely on sites readily accessible to plasma‐derived complement and plasma‐infused complement inhibitors. However, such systemic approaches inevitably result in broad inhibition of the complement cascade. Given the central role of complement in host defense, this global suppression can increase susceptibility to infections and other immune‐related complications. Consequently, therapeutic strategies that enable targeted modulation of complement dysregulation directly at sites of pathology may provide a more precise approach, preventing complement‐driven damage while preserving systemic immune function. In addition, gaining access to tissues not readily reached via the plasma will open up new possibilities for complement intervention.

Effective penetration into tissues in which complement activation is driven predominantly by locally produced complement may, however, be more challenging. Importantly, in these conditions, one of the earliest host responses is inflammation, following the release of the anaphylatoxins C3a and C5a. This inflammatory response induces vasodilation and increased vascular permeability [129], allowing fluid phase and plasma‐derived proteins to enter affected tissues. Such conditions provide an opportunity for therapeutics to access otherwise restricted sites. Given that complement‐inhibitory treatments are administered therapeutically rather than prophylactically, it is reasonable to assume that intravenously administered drugs can reach inflamed tissues. Moreover, the size and molecular composition will also influence tissue penetration. While smaller molecules are generally expected to exhibit improved tissue diffusion, immunohistological analyses of inflamed tissues frequently reveal the presence of large plasma proteins such as IgM and C1q, indicating that even high‐molecular‐weight proteins can gain access under inflammatory conditions.

Accessing the diseased tissue is the first step, followed by engagement of the appropriate molecular target. There is still much to learn to optimize strategies to enable locally targeted, precision inhibition and to appreciate the differences with currently employed systemic approaches.

In addition to local drug administration, as exemplified by therapies for AMD‐GA, novel targeting strategies have emerged that guide therapeutics to sites of complement dysregulation. Two main design principles have been pursued in their conceptualization and have shown promising effects in in vitro settings and early stages of preclinical investigations. First, therapeutic molecules can be directed toward complement‐independent but disease‐specific surface antigens (reviewed in [13]). Second, covalently deposited late‐stage complement opsonins may serve as docking sites for the targeting moiety of the therapeutic (reviewed in [130]). Various molecular modalities have been used as the basis for this targeting unit, including natural antibodies (e.g., [131, 132]), bispecific antibodies (e.g., [133, 134]), or single‐chain antibodies (e.g., [135]). To target sites of complement activation, either anti‐C3d antibodies are used or functional domains from complement receptors that naturally bind to C3 fragments (e.g., [136, 137]).

**FIGURE 3:**
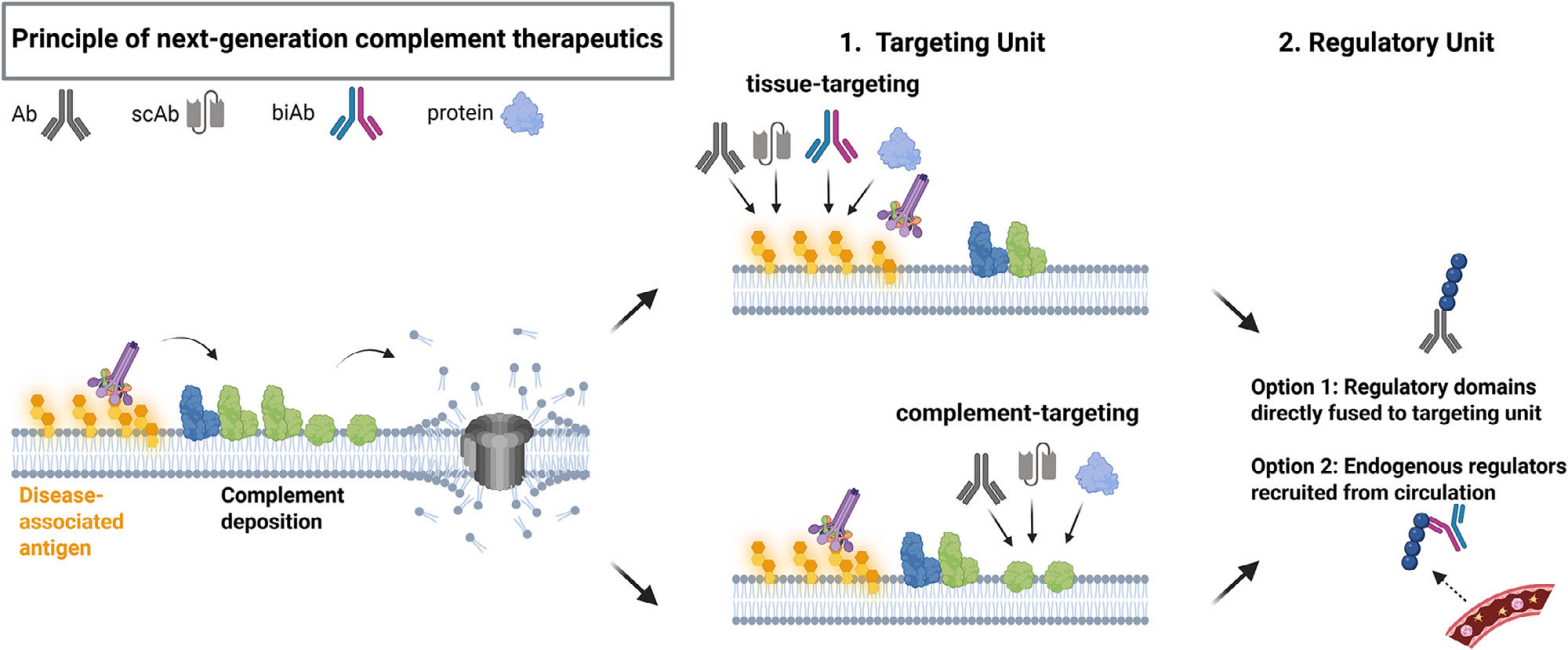
Principle of next‐generation complement therapeutics. Complement therapeutics consist of two essential units: a targeting unit and a regulatory unit. The targeting unit can be directed either against a disease‐specific epitope or against a degraded complement activation fragment. The regulatory unit provides the therapeutic effector function and can either be directly fused to the targeting unit or be recruited from circulation. Ab: antibody, scAb: single‐chain antibody; biAb: bispecific antibody.

Beyond the targeting unit, both approaches share the common feature that the complement‐interfering component consists of full‐length or functionally active domains derived from naturally occurring complement regulators. These regulatory elements may either be directly fused to the targeting moiety or recruited from circulation (Figure 3). For more detailed information on currently investigated modalities based on these principles, we refer to two recent comprehensive reviews [13, 130].

## 8 Opportunities and Hurdles of Next‐Generation Complement Therapeutics

These emerging concepts hold substantial promise considering the above‐mentioned limitations of existing therapies with currently approved inhibitors that act systemically and rely on stoichiometric target engagement. If tissue‐specific delivery and stable target binding can be achieved, several important advantages may arise.

First, required drug concentrations may be reduced, as inhibitors would be locally confined and active at the site of disease while largely preserving systemic complement activity. Therapeutic efficacy may benefit from avidity effects resulting from high target antigen density or extensive opsonin deposition, thereby retaining the inhibitor within affected tissues. Second, such localization could preserve physiological complement function and reduce the infection risk. Therefore, it is critical to identify and define unique molecular disease patterns that can be therapeutically targeted. One example is the identification of autoantibodies against carbamylated proteins, which drive early rheumatoid arthritis pathogenesis and predict clinical disease onset [138, 139]. This discovery now forms the basis for the targeting moiety of bispecific antibodies in the current development for the treatment of RA [133]. Another example is the antibody‐based targeting of C3d opsonized target cells that has been optimized [140] and now tested in the context of aHUS [141].

Third, regulatory complement inhibitors operate through mechanisms that more closely resemble physiological regulation. Rather than mediating stoichiometric blockade, these molecules function in an enzyme‐like manner, enabling sequential interactions with multiple target molecules following completion of a regulatory event. In a comprehensive side‐by‐side in vitro comparison, regulatory intervention was effective at substantially lower concentrations than stoichiometric inhibitors, which require concentrations exceeding plasma levels of their targets to achieve inhibition [142].

Importantly, both the mode of action and rate of administration are expected to substantially influence therapeutic efficiency. For example, antibody‐based complement inhibitors carrying FH, when administered at high doses, may primarily inhibit the complement systemically, similar to currently available inhibitors, thereby increasing infection risk. In contrast, administration at lower concentrations over extended periods of time may promote accumulation at target sites, resulting in strong local complement inhibition while avoiding any inhibitory effect on the systemic complement activity. Especially modalities that recruit endogenous regulators from circulation require careful dosing strategies to avoid depletion, which could otherwise induce complement‐mediated complications. Given the high physiological plasma concentrations of FH and the anticipated efficiency of local targeting, the risk appears manageable; nevertheless, careful design of intervention studies will be essential. The extent to which all these assumptions translate to the in vivo situation remains to be determined. For many next‐generation fusion constructs, pharmacodynamic and pharmacokinetic profiles remain insufficiently characterized [130]. Moreover, each disease context and target tissue imposes distinct constraints, necessitating careful evaluation of which pathological settings are most suitable for these approaches and which molecular designs are optimal. For example, targeting long‐lived matrix components may require fundamentally different strategies compared with targeting erythrocyte surfaces, which exhibit considerably shorter lifespans.

Next‐generation complement inhibitors designed to home to complement‐opsonized surfaces present additional challenges. While efficient targeting of diseased tissues is expected, these agents may also bind to opsonized pathogens, potentially providing unintended protection and thereby increasing susceptibility to infection. To date, no clear infectious complications have been reported; however, continued monitoring will be crucial.

As with all biological therapeutics, next‐generation complement inhibitors may also face immunogenicity challenges. The closer an inhibitor resembles naturally occurring proteins, the lower the likelihood of eliciting immune responses. Genetically engineered fusion constructs, such as antibody‐FH fusion proteins, inevitably contain artificial domain combinations and linker sequences that may introduce immunogenic epitopes. In contrast, bispecific human antibodies that recruit endogenous FH are expected to exhibit lower immunogenic potential, although experimental data addressing this issue remain limited.

Taken together, next‐generation complement inhibitors hold significant potential to overcome several limitations associated with currently approved therapies. At the same time, these emerging strategies introduce new scientific and translational challenges that need to be addressed.

## 9 Complement Restoring and Activating Therapeutics

Although complement overactivation is associated with many human diseases, it is important to recognize that potent effector functions of complement can also be harnessed therapeutically, particularly to combat infections or cancer.

In a nonspecific manner, this concept has already been realized in patients suffering from genetic complement deficiencies [143, 144, 145]. For example, C2‐deficient patients with SLE receiving repeated plasma infusions experienced complete remission of the disease phenotype and restoration of hemolytic complement activity [146]. In another case, a patient with an SLE‐like disease with dysfunctional C3 showed correction of complement function following plasma infusion, as evidenced by solubilization of immune complexes [147]. These observations demonstrate that plasma infusions can reconstitute missing or dysfunctional complement components. However, plasma infusions also carry potential risks [148], including allergic or anaphylactic reactions and transfusion‐related injuries (e.g., acute lung injury [149]).

While these approaches aim to restore complement activity, the powerful functions can also be intentionally exploited for therapeutic benefit. A prominent example is cancer immunotherapy. Antibody‐based cancer immunotherapies can broadly be divided into two categories. The first includes mAbs that modulate cellular immune functions, such as those that block immune checkpoints (e.g., PD‐1‐PD‐L1 pathway) or that cross‐link tumor cells and T cells through T‐cell‐engaging antibodies [150, 151]. The second category includes antibodies that bind directly to tumor cells and mediate tumor cell killing through mechanisms such as antibody‐dependent cellular cytotoxicity (ADCC), antibody‐dependent cellular phagocytosis (ADCP), or complement‐dependent cytotoxicity (CDC) [152]. The design requirements for these two classes of mAbs differ substantially. For checkpoint inhibitors and T‐cell engagers, complement activation and killing of effector immune cells must be avoided, while for antibodies that directly bind the target tumor cells (and induce CDC), complement activation should ideally be optimized.

Modern mAb engineering provides numerous opportunities to tailor therapeutic antibodies to achieve these desired functional properties. For example, checkpoint inhibitors and T‐cell engagers can incorporate Fc‐silencing mutations such as the well‐known LALAPG mutation (L234A/L235A/P329G), which prevents interactions with cellular Fcy receptors and reduces the binding of C1q, thereby minimizing complement activation [153]. In contrast, complement‐activating antitumor antibodies may be used in their wild‐type format or can be further optimized for complement activation by introducing mutations that promote on‐target antibody hexamerization [154]. For several widely used therapeutic antibodies, including rituximab, daratumumab, and alemtuzumab, complement activation is known to represent a major effector mechanism [152]. In some cases, complement activation can be so extensive that complement components become depleted, and, again, supplementation with fresh frozen plasma (FFP) has been proposed to restore complement activity [155].

Numerous strategies are currently being explored to further enhance complement activation beyond the initial antibody‐triggered response [152]. For example, approaches aimed at inhibiting tumor cell‐bound complement regulators [156, 157], or introducing additional complement‐activating molecules [158], enhanced hexamerization [159], or hexamers comprising two antibody reactivities [160] are being tested in in vitro and in preclinical models to improve complement‐mediated tumor cell killing.

Like complement‐inhibitory therapies, complement‐activating strategies must also achieve tissue and cell‐type specificity. Complement‐enhancing effects should ideally be directed to a unique target site, avoid systemic activation, sink effects, and unintended inflammatory responses.

## 10 Conclusion

Over the past two decades, major advances have clarified how dysregulated complement activation contributes to the pathogenesis of a wide range of human diseases, and clinical success with complement inhibitors has firmly established complement as a tractable therapeutic target. The next challenge for the field is to move beyond systemic blockade toward strategies that modulate complement activity directly at the sites of the disease. Such local inhibitors have the potential to improve safety by preserving systemic complement function while suppressing pathogenic complement activation within affected tissues. In addition, site‐directed therapies may enable lower drug doses, reduce treatment costs, and ultimately broaden the clinical accessibility of complement‐targeted interventions across a wider spectrum of diseases.

## Author Contributions

Marco Mannes and Leendert A. Trouw conceived the study. Marco Mannes, Wioleta M. Zelek, and Leendert A. Trouw wrote the manuscript.

## Funding

Wioleta M. Zelek is supported by the UKRI Future Leaders Fellowship Award [UKRI2337].

## Conflicts of Interest

Leendert A. Trouw is listed as an inventor on a patent describing the use of bispecific antibodies for complement inhibition. The remaining authors declare no conflicts of interest.

## Data Availability

No new data were created or analyzed in this study. Data sharing does not apply to this article.
